# Effect of the types of pretreatment imaging modalities on the treatment response to palliative radiation for painful bone metastases from solid cancer: a single-center retrospective analysis

**DOI:** 10.1186/s13014-019-1310-4

**Published:** 2019-06-07

**Authors:** Yuki Wada, Akira Anbai, Satoshi Kumagai, Eriko Okuyama, Kento Hatakeyama, Noriko Takagi, Manabu Hashimoto

**Affiliations:** Department of Radiology, Akita University Graduate School of Medicine, 1-1-1 Hondo, Akita, Akita 010-8545 Japan

**Keywords:** Palliative radiation, Imaging modality, Bone metastasis

## Abstract

**Background:**

Determining the appropriate gross tumor volume is important for irradiation planning in addition to palliative radiation for bone metastases. While irradiation planning is commonly performed using simulation computed tomography (CT), magnetic resonance imaging (MRI), bone scintigraphy, and ^18^fluorodeoxyglucose-positron emission tomography-CT (^18^FDG-PET-CT) are more sensitive for detecting bone metastasis and invasion areas. Therefore, this study evaluated whether pretreatment imaging modalities influenced the response to palliative radiation therapy (i.e., the irradiation effect) for painful bone metastases from solid malignant carcinomas.

**Methods:**

Consecutive patients with painful bone metastases treated with palliative radiation between January 2013 and December 2017 at our institution were included. We retrospectively investigated the pretreatment images from the different imaging modalities (CT, MRI, bone scintigraphy, and ^18^FDG-PET-CT) obtained between 1 month before and the initiation of palliative radiation and determined the primary site of carcinoma, histological type, metastatic lesion type (osteolytic, osteoblastic, or mixed), pathological fracture, and metastatic site (vertebral or not). We then evaluated the relationship between these factors and treatment response. We defined “response” as the condition in which patients achieved pain relief or reduced the use of painkiller medicines.

**Results:**

In total, 131 patients (78 men and 53 women) were included; the median age was 66 years (range, 24–89 years). Prescribed doses were 8–50 Gy/1–25 fractions with 2–8 Gy/fraction. Among the 131 patients, 105 were responders (response rate, 80%). The imaging modalities performed before irradiation were CT in 131 patients, MRI in 54, bone scintigraphy in 56, and ^18^FDG-PET-CT in 14. The Welch t-test and chi-square test showed no significant association between treatment response and each factor. Multiple logistic regression analysis including the imaging modality, metastatic site, and pathological fracture also showed no significant association with each factor.

**Conclusions:**

There was no significant relationship between the type of pretreatment imaging and treatment response for painful bone metastases. Thus, setting the appropriate radiation field according to CT images and clinical findings could help avoiding further image inspection before palliative radiation for painful bone metastases.

## Background

Bone metastases cause functional impairment and severe pain and worsen the quality of life of patients with advanced cancer. Palliative radiotherapy (RT) is useful and effective treatment choice for symptomatic bone metastases [1, 2]. Most patients with symptomatic bone metastases tend to have short survival times; therefore, palliative RT should be started as soon as possible, and physicians should consider using hypofractionated RT regimens such as 8 Gy/fr [3].

In Japan, three-dimensional conformal radiotherapy (3D-CRT) with contouring target volume based on simulation computed tomography (CT) is the most commonly used treatment planning method, as stereotactic body radiotherapy (SBRT) for bone metastasis is not covered by the Japanese insurance system. Studies have shown the usefulness of using magnetic resonance imaging (MRI) scans to determine the gross tumor volume (GTV) that should be treated with palliative RT for bone metastases [4, 5]. Accordingly, we believe that MRI is sensitive for determining bone metastatic lesions; however, the effect of palliative RT, especially 3D-CRT, in clinical situations is not well known, especially considering the use of radiation planning with or without MRI or other functional imaging, bone scintigraphy, and ^18^fluorodeoxyglucose-positron emission tomography (^18^FDG-PET)/CT, all of which are sensitive for bone metastases [6, 7]. The addition of further examinations including MRI, bone scintigraphy, and ^18^FDG-PET/CT results in delays in starting RT and increases the cost.

The present study was performed based on the hypothesis that MRI, bone scintigraphy, and ^18^FDG-PET/CT are more sensitive for detecting bone metastatic lesions compared to CT; therefore, we believed that using further imaging modalities might improve the response rate of palliative radiation for painful bone metastases. Accordingly, in the present study, we retrospectively analyzed the effect of palliative RT performed with or without MRI, bone scintigraphy, and ^18^FDG-PET/CT among patients with painful bone metastases from solid malignant tumors.

## Methods

### Inclusion and evaluation criteria

The study was approved by the Institutional Review Board of our institution (No. 1914, approved on February 15, 2018). We included all consecutive patients with painful bone metastases from solid malignant tumors treated with palliative RT in our institution between January 2013 and December 2017. We retrospectively analyzed the following characteristics using imaging modalities performed 1 month before the start of RT: primary site of malignancy, histological type of the primary tumor, type of bone metastases based on radiographic appearance [8] (osteolytic, osteoblastic, or mixed), pathological fractures, and the metastatic site (vertebral or not). In the present study, we evaluated images of imaging modalities performed only 1 month before the start of RT because images obtained at ≥2 months before the start of RT might result in an underestimation of the bone lesions due to tumor progression. We included the metastatic site, i.e., whether it was the vertebral bone or not, as a variable because it is usual to contour the whole body of the metastatic vertebra in the clinical target volume (CTV) when treating vertebral bone metastases with the 3D-CRT method; thus, the influence of the GTV on vertebral bone metastases was suspected to be less than that for long or flat bone metastases. We defined “response” when pain relief was confirmed or the use of painkiller medicines was reduced (i.e., the response group). Any other response apart from that observed in the response group was defined as “non-response”.

### Irradiation planning

All radiation plans were obtained using 3D-CRT in which target volumes were contoured on simulation CT images along with the findings of pretreatment diagnostic images and clinical examinations (i.e. location of spontaneous pain, moving pain, and tenderness), generally according to the guidelines of the Japanese Society for Radiation Oncology [9]. In some cases, we placed surface markers such as wires and catheters at the location of existing pain when performing simulation CT for reference of contouring the GTV. Briefly, the GTV was defined as bone metastases observed during physical examination and diagnostic imaging. The CTV was defined as the GTV plus approximately 1 cm, and the planning target volume was defined as the CTV plus approximately 0.5–2 cm. The prescribed dose was 8–50 Gy/1–25 fractions using 6–10 MeV X-rays [9–12], all of which have the same effect reportedly. For determination of the prescribed dose, radiation oncologists considered the patients’ expected survival, performance status, intensity of pain, and other factors.

### Statistical analysis

Significant differences between the response group and the non-response group were determined using the Welch t-test and chi-square test for univariate analysis depending on the data distribution, and using multiple logistic regression analysis for multivariate analysis. For multivariate analysis, we used the imaging modality, pathological fracture, and metastasis site as explanatory variables that were considered to have a treatment effect. A *p*-value of < 0.05 was considered significant. All analyses were performed using the BellCurve for Excel version 2.00 (Tokyo, Japan).

## Results

### Patient characteristics

A total of 131 patients (78 men and 53 women) were included in the present study. Table 1 shows the patient characteristics. The median age was 66 years (range, 24–89 years). The most common primary tumor sites were the lungs (*n* = 35, 27%), genitourinary tract (*n* = 34, 26%), and gastrointestinal tract (*n* = 34, 26%), and the primary histology was adenocarcinoma in ~ 50% of the cases (*n* = 66). The metastatic bone lesions were vertebral in 86 (66%) cases and non-vertebral in 45 (34%), and the types of bone metastatic lesions were osteolytic in 88 (67%), osteoblastic in 34 (26%), and mixed type in 9 (7%) cases. Pathological fractures, evaluated using CT images, were observed in 24 (18%) patients. Among the 131 patients enrolled, a response was observed in 105 patients (80%), according to our response criteria, who were included in the response group.

**Table 1:** Baseline characteristics and treatment effect of all included patients

Age, years; median (range)	66 (24–89)
Observation period, days; median (range)	100 (7–1514)
Sex	
Male	78 (60%)
Female	53 (40%)
Primary site	
Lungs	35 (27%)
Genitourinary tract	34 (26%)
Gastrointestinal tract	34 (26%)
Breasts	6 (5%)
Head and neck	5 (4%)
Thyroid	4 (3%)
Gynecology	4 (3%)
Unknown	9 (7%)
Histology	
Squamous cell carcinoma	9 (7%)
Adenocarcinoma	66 (50%)
Small cell carcinoma	4 (3%)
Clear cell carcinoma	22 (17%)
Others	27 (21%)
Unknown	3 (2%)
Imaging modality	
CT (including simulation CT)	131 (100%)
MRI	54 (41%)
Bone scintigraphy	56 (43%)
^18^FDG-PET	14 (11%)
Metastatic site	
Vertebral	86 (66%)
Non-vertebral	45 (34%)
Bone metastases type	
Osteolytic	88 (67%)
Osteoblastic	34 (26%)
Mixed	9 (7%)
Pathological fracture	
Present	24 (18%)
Absent	107 (82%)
Treatment effect	
Response	105 (80%)
Non-response	26 (20%)

*CT* computed tomography, *MRI* magnetic resonance imaging, ^*18*^*FDG-PET* ^18^fluorodeoxyglucose-positron emission tomography

The images were obtained within 1 month before the start of palliative radiation by using CT (including simulation CT scan for irradiation planning) in 131 patients (100%), MRI in 54 (41%), bone scintigraphy in 56 (43%), and ^18^FDG-PET/CT in 14 (11%). Some of these patients underwent several modalities; 38 patients underwent only CT; 26 underwent CT and MRI; 31 underwent CT and bone scintigraphy; 6 underwent CT and ^18^FDG-PET; 22 underwent CT, MRI, and bone scintigraphy; 5 underwent CT, MRI, and ^18^FDG-PET; 2 underwent CT, bone scintigraphy, and ^18^FDG-PET; and 1 underwent CT, MRI, bone scintigraphy, and ^18^FDG-PET. Table 2 shows the detailed patient characteristics considering each imaging modality.

**Table 2:** Baseline characteristics and treatment effect of patients considering each pretreatment imaging modality (duplication was allowed)

	CT (including simulation CT) (*n* = 131)	MRI (*n* = 54)	Bone scintigraphy (*n* = 56)	^18^FDG-PET (*n* = 14)
Age, years; median (range)	66 (24–89)	67 (35–89)	67 (24–89)	66 (48–84)
Sex				
Male	78 (60%)	34 (63%)	33 (59%)	8 (57%)
Female	53 (40%)	20 (37%)	23 (41%)	6 (43%)
Primary site				
Lungs	35 (27%)	14 (26%)	25 (45%)	4 (29%)
Genitourinary tract	34 (26%)	16 (30%)	10 (18%)	6 (43%)
Gastrointestinal tract	34 (26%)	14 (26%)	14 (25%)	1 (7%)
Breasts	6 (5%)	3 (6%)	2 (4%)	0 (0%)
Head and neck	5 (4%)	3 (6%)	1 (2%)	2 (14%)
Thyroid	4 (3%)	1 (2%)	1 (2%)	0 (0%)
Gynecology	4 (3%)	0 (0%)	0 (0%	0 (0%)
Unknown	9 (7%)	3 (6%)	3 (5%)	1 (7%)
Histology				
Squamous cell carcinoma	9 (7%)	5 (9%)	4 (7%)	2 (14%)
Adenocarcinoma	66 (50%)	25 (46%)	28 (50%)	6 (43%)
Small cell carcinoma	4 (3%)	3 (6%)	4 (7%)	0 (0%)
Clear cell carcinoma	22 (17%)	9 (17%)	11 (20%	1 (7%)
Others	27 (21%)	10 (19%)	7 (13%)	5 (36%)
Unknown	3 (2%)	2 (4%)	2 (4%)	0 (0%)
Metastatic site				
Vertebral	86 (66%)	44 (81%)	38 (68%)	8 (57%)
Non-vertebral	45 (34%)	10 (19%)	18 (32%)	6 (43%)
Bone metastases type				
Osteolytic	88 (67%)	43 (80%)	39 (70%)	9 (64%)
Osteoblastic	34 (26%)	7 (13%)	13 (23%)	4 (29%)
Mixed	9 (7%)	4 (7%)	4 (7%)	1 (7%)
Pathological fracture				
Present	24 (18%)	9 (17%)	9 (16%)	4 (29%)
Absent	107 (82%)	45 (83%)	47 (84%)	10 (71%)
Treatment effect				
Response	105 (80%)	43 (80%)	45 (80%)	12 (86%)
Non-response	26 (20%)	11 (20%)	11 (20%)	2 (14%)

*CT* computed tomography, *MRI* magnetic resonance imaging, ^*18*^*FDG-PET* ^18^fluorodeoxyglucose-positron emission tomography

### Univariate analysis

Table 3 shows the results of univariate analysis between the response group and the non-response group. On univariate analysis, the pretreatment imaging modality did not show any significant relationship with the treatment effect; the *p*-values for MRI, bone scintigraphy, and ^18^FDG-PET were 0.54, 0.57, and 0.45, respectively. The other factors included in the present study were age (*p* = 0.21), sex (*p* = 0.18), primary site of malignancy (*p* = 0.69), histology of the primary malignancy (*p* = 0.27), metastases site (*p* = 0.57), bone metastases type (*p* = 0.47), and pathological fracture (*p* = 0.21); overall, there was no significant relationship with the treatment effect.

**Table 3:** Univariate analysis of patents divided into the response and non-response groups

	Response group	Non-response group	*p*-value
Age, years; median (range)	67 (35–89)	64 (24–77)	0.21
Sex (Male: Female)	60:45	18:8	0.18
Primary site (Lungs: GU tract: GI tract: Breasts: HN: Thyroid: Gyne: Unknown)	27:26:29:5:4:4:4:6	8:8:5:1:1:0:0:3	0.69
Histology (SCC: Adeno: Small: CC: Others: Unknown)	5:53:4:17:24:2	4:13:0:5:3:1	0.27
Metastases site (Vertebral or non-vertebral)	67:38	19:7	0.57
Bone metastases type (Osteolytic: Osteoblastic: Mixed)	72:25:8	16:9:1	0.47
Pathological fracture (Present: Absent)	17:88	7:19	0.21
MRI	43 (41%)	11 (42%)	0.54
Bone scintigraphy	45 (43%)	11 (42%)	0.57
^18^FDG-PET	12 (11%)	2 (8%)	0.45

*GU* Genitourinary, *GI* gastrointestinal, *HN* head and neck, *Gyne* Gynecology, *SCC* squamous cell carcinoma, *Adeno* adenocarcinoma, *Small* small cell carcinoma, *CC* clear cell carcinoma, *MRI* magnetic resonance imaging, ^*18*^*FDG-PET* ^18^fluorodeoxyglucose-positron emission tomography

### Multivariate analysis

No significant prognostic factor was observed on univariate analysis. Therefore, we performed multivariate analysis with the metastases site, pathological fracture, MRI, bone scintigraphy and ^18^FDG-PET as explanatory variables, which were target factors that might influence the treatment effects. Table 4 shows the results of multivariate analysis; no significant relationship was observed. After adding pathological fracture and metastases site, MRI, bone scintigraphy, and ^18^FDG-PET were not predictive factors for treatment effect of palliative radiation for painful bone metastases.

**Table 4:** Multivariate analysis of patients divided into the response and non-response groups

	Odds ratio	95% CI	*p*-value
Metastases site (Vertebral or non-vertebral)	1.18	0.4482–3.1082	0.74
Pathological fracture	2.06	0.7252–5.8318	0.18
MRI	1.14	0.4553–2.8480	0.78
Bone scintigraphy	0.96	0.3966–2.3427	0.94
^18^FDG-PET	0.56	0.1128–2.8277	0.49

*CI* confidence interval, *MRI* magnetic resonance imaging, ^*18*^*FDG-PET* ^18^fluorodeoxyglucose-positron emission tomography

## Discussion

In the present study, no significant relationship was observed between pretreatment images and treatment effect of palliative radiation for painful bone metastases. The present study was performed based on the hypothesis that MRI, bone scintigraphy, and ^18^FDG-PET/CT are more sensitive for detecting bone metastatic lesions compared to CT; therefore, we believed that using further imaging modalities might improve the response rate of palliative radiation for painful bone metastases. Bone scintigraphy is useful for screening bone metastatic in the whole body; however, its ability to detect early stage bone metastasis is poor [13]. MRI is more sensitive than CT in defining the extent of tumors in the marrow and in soft tissues [14]. MRI is also better for detecting the invasion to the marrow, soft tissues, and joints; however, CT is preferred for evaluating the destruction of the bone cortex [15, 16]. The same study [15] also showed that it is impossible to detect the exact length of malignant lesions in the bone irrespective of the use of CT or MRI. For detecting vertebral metastases, the sensitivity of MRI was 98.5% and that of CT was 66.0% [17]. Bone scintigraphy is also sensitive especially when evaluating systemic bone lesions, although MRI is superior for the diagnosis of local lesions [18]. In fact, The Japanese Imaging Guidelines 2013 [19] recommend using MRI for the diagnosis of malignant bone tumors; however, the same recommendation is not given for CT. Furthermore, ^18^FDG-PET/CT is more sensitive than CT for detecting bone metastatic lesions [7, 13, 20, 21]. ^18^FDG-PET/CT was able to detect bone metastases in 25% of patients who had no abnormal findings on CT images [21]. Recent studies have highlighted the usefulness of whole-body MRI [22] and diffusion-weighted whole-body imaging with background body signal suppression [23].

MRI, bone scintigraphy, and ^18^FDG-PET/CT are superior to CT for detecting bone metastatic lesions. Several studies [4, 5] showed the usefulness of MRI scans for contouring the GTV area for palliative radiation of bone metastases. Among CT, CT-MRI, and MRI-based contouring of the GTV for bone metastases, the use of MRI provided the highest consistency in the GTV area between observers [4]. When CT was used alone and when T1- and T2-weighted MRI was also performed for contouring non-vertebral bone metastases, the addition of T1-weighted images to CT reduced inter-observer variability not only between radiation oncologists but also between radiation oncologists and diagnostic radiologists. However, these studies focused only on contouring the GTV and did not evaluate the treatment effects. Moreover, standardized uptake value of ^18^FDG-PET/CT is useful for predicting the treatment effect of palliative radiation for bone metastasis [24, 25]; however, there is insufficient data for determining the target volume with ^18^FDG-PET/CT.

To our knowledge, no previous study has evaluated whether CT alone or adding more sensitive imaging modalities such as MRI, bone scintigraphy, and ^18^FDG-PET/CT has an effect on the treatment response of palliative radiation for symptomatic bone metastases in a clinical situation. Our study is the first to investigate the treatment effect of different types of pretreatment imaging modalities in a clinical setting. Our results showed that there was no significant relationship between treatment effect and any pretreatment imaging modality. We believe that the reason for these findings is that when we contoured the GTV in clinical settings, we not only considered the imaging findings but also the clinical examination findings and the patient’s reported symptoms. We contoured the GTV as the area where the patients reported pain or tenderness, or where we strongly suspected could be associated with the patient’s symptoms. The microenvironment of metastatic bones is very complex [26], and it is not clear why bone metastases cause pain nor why radiation decreases pain due to bone cancer [27]. In other words, it is not known whether bone metastases cause pain or whether they influence the bone microenvironment, thereby causing pain. One of the reasons for bone metastases pain is a result of the effect of cytokines such as IL-1, IL-4 and TNF-α, all of which are produced during the inflammation process caused by bone metastases; therefore, we should further investigate whether only visible bone lesions cause symptoms or whether invisible factors including cytokines may cause pain.

The present study has several limitations. First, this was a retrospective study; therefore, the treatment response was evaluated based on medical records and the evaluation criteria were not consistent. We defined “response” in the current study as the condition when pain relief was confirmed or the use of painkiller medicines was reduced; however, these criteria were very subjective. Second, patients who met certain criteria were required to undergo further imaging modalities such as MRI, bone scintigraphy, and ^18^FDG-PET. Almost all of the MRIs, bone scintigraphies, and ^18^FDG-PETs were done based on the determination of the attending physician, not by a radiation oncologist. Some patients were required to undergo further imaging modalities requested by a radiation oncologist; however, there were no specific criteria to add/not to add images. In addition, the treatment method, especially considering the prescribed dose, was not uniform. All the included patients were treated with effective doses [10–12]; therefore, the effect of the dose schedule was not important in considering the treatment effect. However, a non-randomized study [28] suggested an increasing need for re-irradiation when we use shorter treatment schedules. We should take both the treatment effect and expected survival into consideration while determining the palliative radiation schedule; therefore, we usually use 30 Gy/10 fr, which is well balanced considering the treatment period and effect. The use of a shorter schedule such as 8 Gy/fr or 20 Gy/4 fr is limited to patients with advanced disease or whose pain is too severe to be treated for long periods.

Despite the several limitations of the present study, our results indicated that MRI, bone scintigraphy, and ^18^FDG-PET/CT might be unnecessary for palliative RT for painful bone metastases. Furthermore, SBRT is not used to treat painful bone metastases, as it is not covered by the insurance system in Japan; however, SBRT is effective and has a shorter treatment schedule compared to 3D-CRT, as determined by studies performed in European and American countries. In the near future, SBRT may also be allowed in Japan considering the evidence published overseas. However, high-precision RT, including SBRT and intensity-modulated RT, has a risk in that the prescribed dose may not cover the “unexpected area,” which has been irradiated by X-rays through the target in conventional 3D-CRT. Therefore, further investigations are needed to determine whether CT is efficient or if MRI is needed when SBRT is performed for bone metastases, as well as to evaluate whether the lesion visible on CT or MRI is present in all malignant sites that show symptoms.

## Conclusions

For preparing the RT plan, there was no significant difference in the response of palliative RT for painful bone metastases with/without the use of MRI, bone scintigraphy, or ^18^FDG-PET/CT. Our results suggest that radiation oncologists can determine the GTV effectively based on CT and clinical examination findings; moreover, it is not necessary to add further imaging examinations before palliative RT when we use 3D-CRT planning.

## Data Availability

None.

## Abbreviations

3D-CRT: Three-dimensional conformal radiotherapy
CT: Computed tomography
CTV: Clinical target volume
FDG: Fluorodeoxyglucose
GTV: Gross tumor volume
MRI: Magnetic resonance imaging
PET: Positron emission tomography
RT: Radiotherapy
SBRT: Stereotactic body radiotherapy
